# Benthic macroinvertebrate field sampling effort required to produce a sample adequate for the assessment of rivers and streams of Neuquén Province, Argentina

**DOI:** 10.1016/j.limno.2017.05.004

**Published:** 2017-07

**Authors:** Joseph E. Flotemersch, Julieta Muñiz Saavedra, Lorena Laffitte, Betina Laurenzano, Marisol Abelli Bonardi, Karen A. Blocksom

**Affiliations:** a U.S. Environmental Protection Agency, Office of Research and Development, 26 W. Martin Luther King Drive, Cincinnati, OH 45268, USA; b Dirección General de Biología Acuática, Neuquén Province, San Martín de los Andes, 8370, Argentina,; c Dirección Provincial de Recursos Hídricos, Neuquén Province, Neuquén 8300, Argentina; d U.S. Environmental Protection Agency, Office of Research and Development, 200 S.W. 35th Street, Corvallis, OR 97333, USA

**Keywords:** Benthic macroinvertebrates, Condition assessment, Sampling, Field method, Target sample size, Neuquén Province, Argentina

## Abstract

This multi-year pilot study evaluated a proposed field method for its effectiveness in the collection of a benthic macroinvertebrate sample adequate for use in the condition assessment of streams and rivers in the Neuquén Province, Argentina. A total of 13 sites, distributed across three rivers, were sampled. At each site, benthic macroinvertebrates were collected at 11 transects. Each sample was processed independently in the field and laboratory. Based on a literature review and resource considerations, the collection of 300 organisms (minimum) at each site was determined to be necessary to support a robust condition assessment, and therefore, selected as the criterion for judging the adequacy of the method. This targeted number of organisms was collected at all sites, at a minimum, when collections from all 11 transects were combined. Subsequent bootstrapping analysis of data was used to estimate whether collecting at fewer transects would reach the minimum target number of organisms for all sites. In a subset of sites, the total number of organisms frequently fell below the target when fewer than 11 transects collections were combined.Site conditions where < 300 organisms might be collected are discussed. These preliminary results suggest that the proposed field method results in a sample that is adequate for robust condition assessment of the rivers and streams of interest. When data become available from a broader range of sites, the adequacy of the field method should be reassessed.

## 1. Introduction

Effective management of riverine ecosystems requires the assessment and evaluation of river condition, using surveys and other direct measures, to determine the anthropogenic impacts to ecosystem structure and function (Parsons et al., 2016). The development of river assessment and monitoring programs has been described in a number of publications (e.g. Bonada et al., 2006; Cairns and Pratt, 1993; Friberg et al., 2011; Parsons et al., 2016). River assessment programs range in complexity from simple programs that focus exclusively on a single element (e.g., water quality/chemistry) to integrated assessment programs that monitor a suite of elements, such as water chemistry, physical habitat, and biological assemblages (Davies et al., 2012; Heiskanen et al., 2004; USEPA, 2011a,b). In general, river assessment commonly includes some type of monitoring mandated as part of government programs or legislation (Lindenmayer and Likens, 2010). Mandated monitoring tracks biological, chemical, hydrological, and/or physical elements of river ecosystems through time to determine trends in river condition and detect environmental harm. This information is, in turn, used by resource managers to effectively manage the riverine systems.

The elements selected in any riverine monitoring program are generally chosen because they change, in some way, in response to anthropogenic impacts and therefore, can be used to infer deterioration or improvement in the condition of the river ecosystem (Downes et al., 2002). Biological assemblages are the central focus of many assessment and monitoring programs, as they provide a direct measure of biological condition relative to biological integrity—a stated objective of, for example, the Clean Water Act of 1972 (33 U.S.C. § 1251 et seq.) and the Water Framework Directive of the European Union (2000/60/EG, Abl. L 327 of 22.12.2000). In addition, biological assessments contribute to narrative water quality standards that are an important part of U.S. state water-laws, and similarly, are essential for enforcement of the U.S. Endangered Species Act (16 U.S.C. § 1531–1544), Canada’s Species at Risk Act (S.C. 2002, c. 29), and the European Union Habitats Directive (92/43/EEC, Abl. L 43 of 21.05.1992). Biota integrate the effects of multiple stressors in space and time (Rosenberg and Resh, 1993); therefore, these sentinels provide a way of detecting environmental stressors that may be so variable in time (e.g., pulses of metal effluent associated with storms) or space (e.g., bank erosion) that they are neither logistically nor economically feasible to monitor directly. A variety of organisms have been used for biological monitoring (e.g., Bonada et al., 2006; Flotemersch et al., 2006; Friberg et al., 2011), but the three most common are algae, macroinvertebrates, and fishes.

Globally, benthic macroinvertebrates are by far the most widely used assemblage in biological monitoring programs, for a host of reasons (Southerland and Stribling, 1995). Benthic macroinvertebrates are the primary consumers in most systems and are an important link between primary resources and higher trophic levels, including many important recreational and commercial fish. Most macroinvertebrates are relatively sessile, which means they are excellent for evaluating site-specific impacts, and collection methods are relatively easy, straightforward, and inexpensive. Macroinvertebrates have a variety of life cycles (i.e., have both short-lived and long-lived taxa), and thus, provide a way of integrating impacts over a variety of time scales (Rosenberg and Resh, 1993). Macroinvertebrates are relatively easy to identify to the family level, and many are easy to identify to genus. Macroinvertebrate taxa vary in their tolerance to different stressors, providing information for interpreting cumulative stressor impacts through community assemblage structure (Rosenberg and Resh, 1993).

When considering methods for sampling benthic macroinvertebrates in flowing waters, those selected should be clear, consistent, reproducible, and most importantly, should effectively support the program(s) for which the data is being collected. They should also perform well across the range of habitats and river types that will be encountered, represent site conditions accurately, and ideally, identify the presence of stressors. Protocols should also be cost-effective, logistically-feasible with only moderate training, and able to meet or be adaptable to multi-purpose sampling needs of researchers and managers (e.g., trend analysis, point source and nonpoint source programs, habitat rehabilitation and restoration efforts, etc.).

Many factors can skew assessment results (Flotemersch et al., 2006; Stribling, 2011), including factors that influence how well field samples represent actual site conditions. For example, a field sampling technique that does not adequately sample benthic macroinvertebrates from across the range of habitats occurring at a site may not adequately reflect actual overall conditions of the site, but rather only conditions of those habitats sampled (Barbour et al., 2006; Blocksom and Flotemersch, 2005). Similarly, a naïve sampling effort can result in the collection of an insufficient number of organisms to support the robust characterization of the benthic macroinvertebrate diversity at a site. To help prevent this, sample size requirements are often established for-field collection efforts. Often, a sample containing more organisms than the target sample size is collected. In that case, samples may be subsampled in the laboratory (i.e., examination and analysis of a subset of the larger composite field sample). This approach has multiple benefits. First, it is an effective tool for conserving resources (Barbour and Gerritsen, 1996; Chen et al., 2015; Flotemersch et al., 2006; Growns et al., 1997; Somers et al., 1998; Vinson and Hawkins, 1996). Second, subsampling helps control for some of the variability across samples due to things other than conditions (e.g., patchiness of habitats, differences in sampling effort, actual differences in the community).

Many studies have recommended laboratory sub-sampling sizes that range from 50 to 500 organisms (Buss et al., 2015; Chen et al., 2015); however, a one-size-fits-all sample size should not be expected, because the information required by researchers and managers can vary depending on individual study needs (Doberstein et al., 2000). But once a sample size has been established, it should be adhered to once an index based on that sample size has been calibrated. In cases where a target laboratory sub-sampling size has been identified, it is important that field sampling protocols be structured to meet these requirements. This, however, is not always possible, especially at sites where productivity is naturally low or at sites impacted by the presence of anthropogenic stressors. These types of sites should be considered exceptions to the rule, and thus part of the assessment interpretation. They should not drive any aspect of the methods development for the larger population of sites. One methods to acknowledge and practically deal with the occurrence of such sites is to set a goal of collecting the target number of organisms at a certain percentage of sites (e.g., Hughes et al., 2002).

The purpose of this pilot study was to evaluate the adequacy of a benthic macroinvertebrate sampling method being considered for use in supporting the assessment of streams and rivers in the Neuquén Province of Argentina. More specifically, the study aimed to answer the question—does the proposed benthic macroinvertebrate field method result in a sample that collected the number of organisms targeted by the program?

## 2. Methods

### 2.1. Study area

**Fig. 1.**
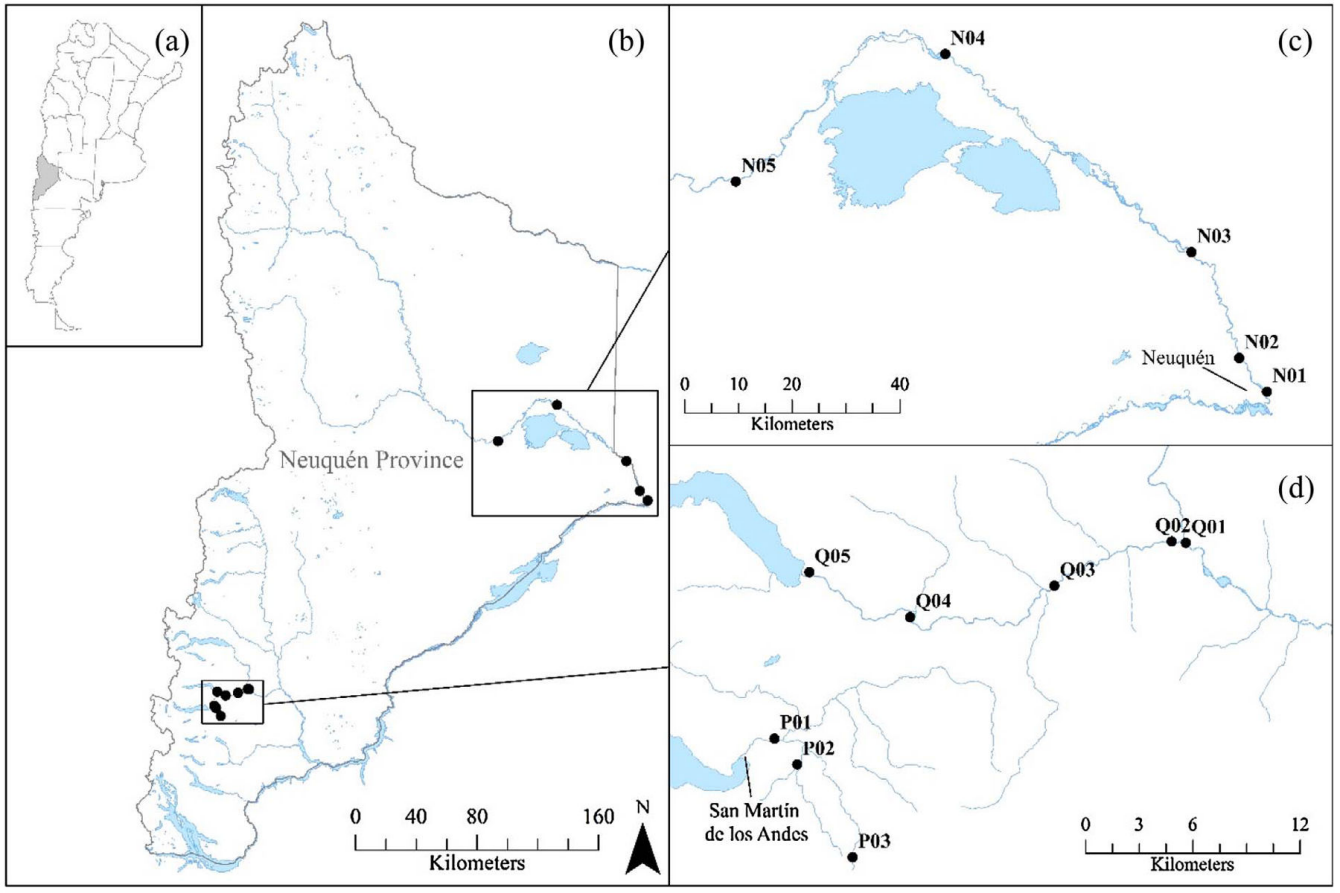
Neuquén Province study sites. (a) Location of the Neuquén Province in Argentina, (b) general location of study sites within the Province, (c) Neuquén River system (N) sites, (d) Quilquihue River system (Q) and Pocahullo River system (P) sites.

A total of 13 sites were sampled, representing a range of conditions that might be encountered in the Neuquén Province of Argentina (Fig. 1, Table 1). Five sites were in the Neuquén River Basin, which has an area of 50,774 km^2^. The Neuquén River meets the Limay River near Neuquén City to form the Río Negro, which continues its way east to the Atlantic Ocean. All Neuquén River system sites were located in the Monte Austral, or “Southern Mountains,” ecological region of the Neuquén Province, which is characterized by mountain ranges, mountains, and hills and is crossed by numerous rivers and streams. The main economic activity in this region is cattle and sheep farming. Others include forest plantations and red deer hunting (Bran et al., 2002). Sample sites in the Neuquén River system were generally lower gradient, non-wadeable rivers with moderate to high levels of anthropogenic impact and substrates generally composed of gravel and sand.

Five sites were in the Quilquihue River Basin, which has an area of 730 km^2^. The Quilquihue River is one of the main tributaries of the Chimehuin River, which flows into the Collon Cura, and then the Limay River. All Quilquihue sites were located in the Precordillera, or “Foothills,” ecological region of the Neuquén Province. This ecological region is similar in characteristics to the Monte Austral ecological region; however, land ownership in this region is generally private, with an increased presence of private subdivisions and fishing lodges (op. cit.). Sites sampled in the Quilquihue River system were higher gradient, non-wadeable rivers with either cobble, cobble-boulder, or gravel-sand substrates and limited anthropogenic impact.

Three sites were sampled in the Pocahullo River Basin, which has an area of 185 km^2^. Streams of the Pocahullo are fed by glacial run-off and seeps and ultimately drain into Lake Lacar in San Martin de Los Andes. Like the Quilquihue, the Pocahullo sites are also located in the Precordillera ecological region. All sites in this system were fully-wadeable and ranged from moderate to high gradient, with moderate to high levels of anthropogenic impact. Substrates at these sites ranged from cobble-gravel at the upper-most site, to sand- and silt-impacted gravel-cobble substrates at the lower two sites. Sites in the Quilquihue and Neuquén River systems were sampled in January 2013; sites in the Pocahullo River system were sampled in March 2015.

### 2.2. Field methods

The field method used for collection of benthic macroinvertebrates was adapted from USEPA (2007). Samples were either collected with a D-ring dip net with a bottom edge of 30.5 cm^2^ or a Surber sampler measuring 30.5 cm^2^ × 30.5 cm^2^ (595-μm mesh) based on gear availably. In brief, a benthic macroinvertebrate sample was collected from a single location along each of 11 transects equally-spaced over a distance of 100 m at each site. The stream length of 100 m was used for this study as this was the distance used for existing field sampling protocols. The first transect was randomly located, and then marked with flagging to identify it as the downstream extent of the study reach. From that point, a systematic sampling design was applied to establish 11 transects (USEPA, 2007) within the reach. This design has many desirable features, and as long as the first transect location is selected at random, the remaining transects based on that initial location can be considered random as well (Cochran, 1977). The simplicity of the design makes it easy to execute without mistakes, results in significant time savings in the field, and also results in the drawn sample being spread more evenly over prevailing habitats, and thus the population (Cochran, 1977; Manly, 2001). Critics of systematic sampling designs express the concern that rare habitats may be missed by this approach. It is important to clarify that the objective of this type of sampling is to collect a ‘sample’ of the benthic macroinvertebrate community for use in the assessment of the system. Data resulting from such sampling events should not be confused with an “inventory” of the community at a site (i.e., an assessment of the benthic macroinvertebrate community); although resultant data can certainly supplement inventory efforts.

At sites that were fully wadeable, the transect samples were collected at points that alternated between 25%, 50%, and 75% of the wetted width. At sites that were not fully wadeable, samples were collected along one shoreline at a depth adequate to submerge the entire net frame or Surber sampler, yet considered safe. At each of the 11 transect sampling points, a substrate area of approximately 30.5 × 30.5 cm (930.25 cm^2^) was disturbed for 30 s to dislodge benthic organisms, resulting in a total sample area of 1.02 m^2^ at each site. At locations where the current was sufficient, dislodged organisms were carried by the current into the waiting net or Surber sampler. If the current was insufficient, the sampling net (or Surber sampler) was swept in the area were the substrate was disturbed to capture suspended organisms. Samples from each of the 11 transects were then cleaned of large debris (e.g., rocks, sticks, and leaves) and then preserved in 70% ethyl alcohol for laboratory processing. In the laboratory, all the benthic macroinvertebrates from each transect sample were sorted, counted, and then stored in 70% ethyl alcohol for later taxonomic identification.

### 2.3. Statistical analysis

A review of the available literature on laboratory subsample size requirements (Barbour and Gerritsen, 1996; Buss et al., 2015; Chen et al., 2015; Flotemersch et al., 2006; Growns et al., 1997; Somers et al., 1998; Vinson and Hawkins, 1996), in conjunction with consideration of available resources, led to a management decision to target collection of at least 300 benthic macroinvertebrates at each site. Hence, the proposed field method was evaluated for its ability to support this target sample size (n = 300) at each site. Parametric summary statistics (i.e., total, mean, standard error) were calculated to aid in the interpretation.

To make additional statistical inference, a bootstrap analysis was conducted. Bootstrapping is a non-parametric statistical approach useful for providing an estimate of confidence when parametric statistical approaches may not be appropriate (e.g., limited data). Bootstrapping was performed on the count data for each site separately. The count of organisms from each transect was treated as a separate data point. For each of 1000 runs, 11 data points were randomly sampled with replacement from the set of counts for that site. These 11 data points (representing counts of organisms collected at transects 1–11) were then added to one another to determine the total number of organisms collected at the site by sampling from 1 to 11 transects, noting the transect number at which, on average, the target sample size (n = 300) was achieved. This process was repeated for each site. This approach is intended to reduce uncertainty surrounding the assumption that the set of random samples at each site represents the true distribution of organisms at that site. Thus, if we see patterns across sites, (e.g., that 8 transects almost always produces a sample containing 300 organisms) we can extend the results to other, as yet, unsampled sites. Bootstrapping results were also evaluated to identify the proportion of runs for each site in which the target sample size (n = 300) was attained.

## 3. Results

Sampling 11 transects at each of the 13 sites in this study resulted in a total of 143 samples, each of which was independently-processed in the field and laboratory (Table 2). The sampling effort resulted in the collection of a total of 17,098 benthic macroinvertebrates. Across all sites, the mean number of organisms collected across transects and the mean number of organisms collected across sites was 119.57 and 1315.23, respectively. The Pocahullo River had the highest mean total number of organisms per site (n = 2185.30) followed by the Neuquén (n = 1541.20) and the Quilquihue (n = 567.20) Rivers. The target number of organisms (n = 300) was collected at all sample sites, but at a few sites in the Quilquihue River system, the total number of organisms collected was close to this targeted minimum.

The standard error of transect sample sizes was calculated for each site to characterize the within-site variability among transects (Table 2). A lower standard error indicates more homogenous conditions at a site with respect to the number of organisms collected, compared to a site with a higher value. Standard error values were generally lower for Quilquihue River sites than in the Neuquén and Pocahullo river sites.

Bootstrapping analysis conducted to provide an estimate of confidence in these findings resulted in totals across all transects at or above the target number of organisms (n = 300) for 100% of the runs for the Pocahullo and Neuquén River sites, but for only one of the Quilquehue sites (Q1; Table 3). Bootstrapping resulted in totals of at least 300 organisms for over 90% of the runs for Quilquehue sites Q2–Q4, but for little more than 60% of the runs for site Q5.

**Fig. 2.**
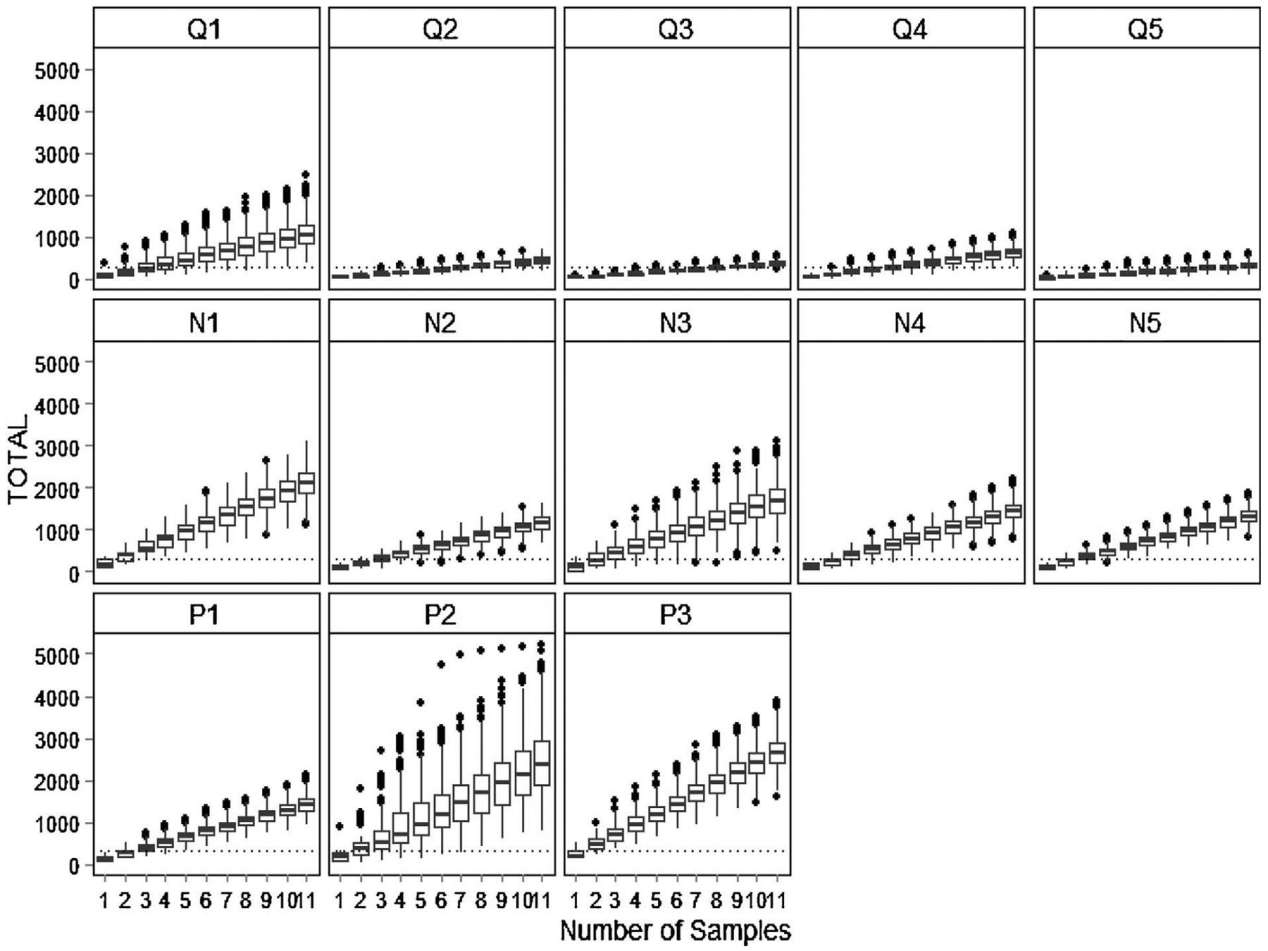
Boxplots showing the full range of sums (total number of benthic macroinvertebrates) for samples at each transect location after 1000 bootstrapping runs. The dotted line indicates the target sample size of 300 organisms.

Graphic representation of the bootstrap analysis (Fig. 2) shows, on average, at what transect the target sample size (n = 300) was achieved. For the Pocahullo and Neuquén River system sites, this was generally after the second or third transect. More transects were required for the sites of the Quilquehue River system, with data from one site suggesting that all eleven transects would need to be sampled.

## 4. Discussion

This research was conducted to determine if the proposed benthic macroinvertebrate field method result in a sample that collected the number of organisms targeted. Based on a review of the available literature and resource considerations, a target sample size of 300 organisms per site was selected as the criterion for judging the adequacy of the method. This should not be misinterpreted to mean that the method would only be considered adequate if it collected ≥300 organisms at every site, but rather that it should result in the target number of organisms at the majority of sites sampled. The targeted number of organisms was collected at each of the 13 sites sampled, when the totals from each site’s 11 transects were summed.

In the bootstrap analysis conducted to estimate confidence of the preceding findings, some sites did not achieve the target sample size (n = 300). This would seem to indicate that it might be likely to collect fewer than 300 organisms at some sites using this field method. There are several reasons why this might happen. The site could have a naturally low density of organisms (e.g., Anderson and Day, 1986), or it could be in very poor condition (e.g., impaired to the extent that it does not even support highly-tolerant organisms). Alternatively, the site could simply be difficult to sample because of the stream bottom habitat. This was the situation encountered at several of the sites sampled in this study. Multiple sites had substrates composed of cobble and boulders, thus making sampling with a net frame or Surber sampler very difficult. In situations where the physical conditions of a site are not conducive to using this field method, one option could be to use a different field sampling method at that site. However, harmonizing data collected using two or more methods has many difficulties associated with it (Cao and Hawkins, 2011 and references cited therein). Beyond the associated data complications, field crews would also have to be trained on multiple methods, carry the gear necessary to execute multiple methods, and likely have to make a decision in the field about which method to use. For these reasons, study designs utilizing multiple sampling methods should be avoided when possible.

Acknowledging that the Neuquén Province contains many streams and rivers with substrates that will make them difficult to sample, we recommend that the language of the final field method include explicit language on how to effectively sample these sites. For example:

When sites are encountered that contain large substrate that make net or sampler placement at any or all transects difficult, a search should be conducted in the immediate area of the transect(s) to locate a suitable location for sample collection. In some streams, it may be necessary to move larger substrates to facilitate satisfactory net placement.

Field sheets should include notes of any sampling difficulties encountered and also include adequate documentation of what method adjustments were necessary to collect a sample adequate for characterization of the site.

## 5. Conclusions

Results of this pilot study support the finding that the proposed field method will result in the collection of a sample that collected the number of organisms targeted by the program (i.e., that meets the target of 300 organisms). However, these results are based on the analysis of only 13 sites. As more data become available from a broader range of sites, the adequacy of the sampling method should be reevaluated. If an unacceptable number of samples (e.g., > 15%) are found to contain an insufficient number of organisms for robust condition assessment (Hughes et al., 2002), the sampling protocol may need to be adjusted to increase the total area sampled in the field. For example, this could be accomplished by increasing the number of samples collected per transect at each site.

## Figures and Tables

**Table 1:** General information about the Neuquén River system (N), Quilquehue River system (Q), and Pocahullo River system (P) sites sampled in this study.

Site ID	Latitude	Longitude	Sample date	Elevation (m)	Average width (m)	Generalized substrate
Ql	40° 3’33.00”	71° 3’ 58.50”	1/24/2013	734.3	17	Cobble
Q2	40° 3’ 27.00”	71° 4’ 39.40”	1/24/2013	741.1	50	Cobble-boulder
Q3	40° 4’ 47.30”	71° 9’ 19.40”	1/24/2013	786.2	35	Cobble
Q4	40° 5’ 37.40”	71° 15’ 1.60”	1/24/2013	861.8	20	Cobble-boulder
Q5	40° 4’ 11.60”	71° 18’ 55.20”	1/23/2013	906.6	45	Gravel-sand
Nl	38° 57’ 35.38”	68° 0’ 26.64”	1/30/2013	258.9	30	Gravel
N2	38° 54’ 15.45”	68° 4’ 2.45”	1/30/2013	275.2	60	Gravel
N3	38° 43’ 41.61”	68° 10’ 18.14”	1/30/2013	302.2	70	Gravel-cobble
N4	38° 24’ 0.78”	68° 42’ 0.78”	1/30/2013	382.7	10	Cobble-boulder
N5	38° 36’ 48.65”	69° 8’ 43.90”	1/29/2013	463.4	60	Silt
PI	40° 9’ 10.00”	71° 20’ 27.40”	3/10/2015	661.8	4.5	Cobble-boulder-gravel
P2	40° 9’ 57.87”	71° 19’ 35.57”	3/10/2015	855.2	3.5	Silt-sand
P3	40° 12’ 48.00”	71° 17’ 30.30”	3/10/2015	1701.7	0.7	Cobble

**Table 2:** Summary of benthic macroinvertebrate collection at the 13 sample sites.

Site	No. organisms per transect											Total no. organisms	Transect	
	1	2	3	4	5	6	7	8	9	10	11		Mean	S.E.
Ql	91	64	40	34	368	29	118	82	13	147	95	1081	98.37	98.33
Q2	79	73	15	86	29	21	24	30	20	43	14	434	39.45	27.0
Q3	21	17	11	28	44	40	34	42	29	27	78	371	33.7	18.0
Q4	49	36	50	7	29	80	35	91	91	10	150	628	57.1	42.5
Q5	72	8	8	21	32	14	36	36	2	85	8	322	29.3	27.3
Grand total and mean across transects for Quilquihue sites												2836	51.4	
Nl	93	117	96	139	220	318	305	90	316	331	*77*	2102	191.1	107.3
N2	157	153	59	134	144	25	106	61	184	108	30	1161	105.6	54.5
N3	369	130	122	27	38	27	112	87	207	349	226	1694	154.0	120.8
N4	76	144	101	210	202	95	213	228	86	38	45	1438	130.7	71.3
N5	115	90	217	106	149	194	162	40	65	74	99	1311	119.2	55.3
Grand total and mean across transects for Neuquén sites												7706	140.1	
PI	202	123	252	73	115	84	168	84	95	177	58	1431	130.1	61.6
P2	264	170	328	36	230	160	22	131	86	125	898	2450	222.7	242.3
P3	270	297	181	177	181	309	330	191	502	114	123	2675	243.2	113.3
Grand total and mean across transects for Pocahullo sites												6556	198.7	

**Table 3:** Results of bootstrapping analysis conducted on data from the 13 sample sites.

Site ID	Mean total no. organisms/ 1000 runs	Percent of runs attaining target sample size (n = 300)
Ql	1082	100
Q2	432	93.9
Q3	369	90.5
Q4	626	99.7
Q5	326	60.9
Nl	2119	100
N2	1160	100
N3	1685	100
N4	1444	100
N5	1306	100
PI	1425	100
P2	2429	100
P3	2659	100

## References

[R1] Anderson RV, Day DM, 1986. Predictive quality of macroinvertebrate—habitat associations in lower navigation pools of the Mississippi River. Hydrobiologia 136, 101–112.

[R2] Barbour MT, Gerritsen J, 1996. Subsampling of benthic samples: a defense of the fixed-count method. J. N. Am. Benthol. Soc. 15, 386–391.

[R3] Barbour MT, Stribling JB, Vandonschot PFM, 2006. The multihabitat approach of USEPA’s rapid bioassessment protocols: benthic macroinvertebrates. Limnetica 25 (3), 839–850.

[R4] Blocksom KA, Flotemersch JE, 2005. Comparison of macroinvertebrate sampling methods for nonwadeable streams. Environ. Monit. Assess. 102, 243–262. 15869189 10.1007/s10661-005-6025-3

[R5] Bonada N, Prat N, Resh VH, Statzner B, 2006. Developments in aquatic insect biomonitoring: a comparative analysis of recent approaches. Annu. Rev. Entomol. 51, 495–523. 16332221 10.1146/annurev.ento.51.110104.151124

[R6] Bran D, Ayeza J, Lopez C, 2002. Áreas ecológicas de Neuquén. Informe Técnico Instituto Nacional de Tecnología Agropecuaria (INTA) N° 70–EEA, Bariloche pp9.

[R7] Buss DF, Carlisle DM, Chon TS, Culp J, Harding JS, Keizer-Vlek HE, Robinson WA, Strachan S, Thirion C, Hughes RM, 2015. Stream biomonitoring using macroinvertebrates around the globe: a comparison of large-scale programs. Environ. Monit. Assess. 187, 4132. 25487459 10.1007/s10661-014-4132-8

[R8] Cairns J Jr., Pratt JR, 1993. A history of biological monitoring using benthic macroinvertebrates. In: Rosenberg DM, Resh VH (Eds.), Freshwater Biomonitoring and Benthic Macroinvertebrates. Chapman and Hall, New York, pp. 10–27.

[R9] Cao Y, Hawkins CP, 2011. The comparability of bioassessments: a review of conceptual and methodological issues. J. N. Am. Benthol. Soc. 30, 680–701.

[R10] Chen K, Hughes RM, Wang B, 2015. Effects of fixed-count size on macroinvertebrate richness, site separation, and bioassessment of Chinese monsoonal streams. Ecol. Indic. 53, 162–170.

[R11] Cochran WG, 1977. Sampling Techniques, 3rd ed. John Wiley and Sons, New York.

[R12] Davies PE, Stewardson MJ, Hillman TJ, Roberts JR, Thoms MC, 2012. Sustainable Rivers Audit 2: The Ecological Health of Rivers in the Murray-Darling Basin at the End of the Millennium Drought (2008–2010), vol. 1 Murray-Darling Basin Authority, Canberra, Australia.

[R13] Doberstein CP, Karr JR, Conquest LL, 2000. The effect of fixed-count subsampling on macroinvertebrate biomonitoring in small streams. Freshw. Biol. 44, 355–371.

[R14] Downes BJ, Barmuta LA, Fairweather PG, Faith DP, Keough MJ, Lake PS, Mapstone BD, Quinn GP, 2002. Monitoring Ecological Impacts: Concepts and Practice in Flowing Waters. Cambridge University Press, Cambridge, UK.

[R15] Flotemersch JE, Stribling JB, Paul MJ, 2006. Concepts and Approaches for the Bioassessment of Non-Wadeable Streams and Rivers, EPA/600/R-06/127. U.S. Environmental Protection Agency, Office of Research and Development, Washington, D.C.

[R16] Friberg N, Bonada N, Bradley DC, Dunbar MJ, Edwards FK, Grey J, Hayes RB, Hildrew AG, Lamouroux N, Trimmer M, Woodward G, 2011. Biomonitoring of human impacts in freshwater ecosystems: the good, the bad and the ugly. Adv. Ecol. Res. 44, 1–68.

[R17] Growns JE, Chessman BC, Jackson JE, Ross DG, 1997. Rapid assessment of Australian rivers using macroinvertebrates: cost and efficiency of 6 methods of sample processing. J. N. Am. Benthol. Soc. 16, 682–693.

[R18] Heiskanen A-S, van de Bund W, Cardoso AC, Nõges P, 2004. Towards good ecological status of surface waters in Europe—interpretation and harmonisation of the concept. Water Sci. Technol. 49, 169–177. 15195435

[R19] Hughes RM, Kaufmann PR, Herlihy AT, Intelmann SS, Corbett SC, Arbogast MC, Hjort RC, 2002. Electrofishing distance needed to estimate fish species richness in raftable Oregon rivers. N. Am. J. Fish. Manage. 22, 1229–1240.

[R20] Lindenmayer DB, Likens GE, 2010. Effective Ecological Monitoring. CSIRO Publishing, Clayton, Australia.

[R21] Manly BFJ, 2001. Statistics for Environmental Science and Management. Chapman and Hall/CRC, Boca Raton.

[R22] Parsons M, Thoms MC, Flotemersch J, Reid M, 2016. Monitoring the resilience of rivers as social? ecological systems: a paradigm shift for river assessment in the twenty-first century. In: Gilvear DJ, Greenwood MT, Thoms MC, Wood PJ (Eds.), River Science: Research and Management for the 21st Century. John Wiley & Sons, Ltd., West Sussex, UK, pp. 197–220.

[R23] Freshwater Biomonitoring and Benthic Macroinvertebrates. In: Rosenberg DM, Resh VH (Eds.), Chapman and Hall, New York.

[R24] Somers KM, Reid RA, David SM, 1998. Rapid biological assessments: how many animals are enough? J. N. Am. Benthol. Soc. 17, 348–358.

[R25] Southerland MT, Stribling JB, 1995. Status of biological criteria development and implementation. In: Davis WS, Simon TP (Eds.), Biological Assessment and Criteria: Tools for Water Resource Planning and Decision Making. Lewis Publishers, Boca Raton, pp. 81–96.

[R26] Stribling JB, 2011. Partitioning error sources for quality control and comparability analysis in biological monitoring and assessment. In: Eldin AB (Ed.), Modern Approaches to Quality Control. InTech, Rijeka Croatia, pp. 59–84.

[R27] USEPA, 2007. National Rivers and Streams Assessment Field Operations Manual, EPA-841-B-07–009. U.S. Environmental Protection Agency, Office of Water, Washington, D.C.

[R28] USEPA, 2011a. A Primer on Using Biological Assessments to Support Water Quality Management, EPA 810-R-11–01. U.S. Environmental Protection Agency, Office of Water, Washington, D.C.

[R29] USEPA, 2011b. National Rivers and Streams Assessment Fact Sheet, EPA 941-F-11–001. U.S. Environmental Protection Agency, Office of Water, Washington, D.C.

[R30] Vinson MR, Hawkins CP, 1996. Effects of sampling area and subsampling procedure on comparisons of taxa richness among streams. J. N. Am. Benthol. Soc. 15, 392–399.

